# Prognostic value of baseline metabolic tumor volume and total lesion glycolysis in patients with lymphoma: A meta-analysis

**DOI:** 10.1371/journal.pone.0210224

**Published:** 2019-01-09

**Authors:** Baoping Guo, Xiaohong Tan, Qing Ke, Hong Cen

**Affiliations:** Department of Chemotherapy, Guangxi Cancer Hospital and of Guangxi Medical University Affiliated Cancer Hospital, Nanning, Guangxi, People’s Republic of China; Northwestern University Feinberg School of Medicine, UNITED STATES

## Abstract

Whether baseline metabolic tumor volume (TMTV) and total lesion glycolysis (TLG) measured by FDG-PET/CT affected prognosis of patients with lymphoma was controversial. We searched PubMed, EMBASE and Cochrane to identify studies assessing the effect of baseline TMTV and TLG on the survival of lymphoma patients. Pooled hazard ratios (HR) for overall survival (OS) and progression-free survival (PFS) were calculated, along with 95% confidence intervals (CI). Twenty-seven eligible studies including 2,729 patients were analysed. Patients with high baseline TMTV showed a worse prognosis with an HR of 3.05 (95% CI 2.55–3.64, p<0.00001) for PFS and an HR of 3.07 (95% CI 2.47–3.82, p<0.00001) for OS. Patients with high baseline TLG also showed a worse prognosis with an HR of 3.44 (95% CI 2.37–5.01, p<0.00001) for PFS and an HR of 3.08 (95% CI 1.84–5.16, p<0.00001) for OS. A high baseline TMTV was significantly associated with worse survival in DLBCL patients treated with R-CHOP (OS, pooled HR = 3.52; PFS, pooled HR = 2.93). A high baseline TLG was significantly associated with worse survival in DLBCL patients treated with R-CHOP (OS, pooled HR = 3.06; PFS, pooled HR = 2.93). The negative effect of high baseline TMTV on PFS was demonstrated in HL (pooled HR = 3.89). A high baseline TMTV was significantly associated with worse survival in ENKL patients (OS, pooled HR = 2.24; PFS, pooled HR = 3.25). A high baseline TLG was significantly associated with worse survival in ENKL patients (OS, pooled HR = 2.58; PFS, pooled HR = 2.99). High baseline TMTV or TLG predict significantly worse PFS and OS in patients with lymphoma. Future studies are warranted to explore whether TMTV or TLG could be integrated into various prognostic models for clinical decision making.

## Introduction

Lymphoma continues to be the most common form of hematological malignancy worldwide [1,2]. Lymphoma is a heterogeneous group of biologically and clinically distinct neoplasms and have been historically divided into two distinct categories: non-Hodgkin lymphoma (NHL) and Hodgkin lymphoma (HL) [3]. Although major progress has been made in the treatment of patients with lymphoma, many still fail to achieve a response or subsequently relapse [4]. These patients are not easily identified by existing pretreatment prognostic indexes such as the IPI (international prognostic index), IPS (international prognostic score for Hodgkin lymphoma [HL]), FLIPI (prognostic score for follicular lymphoma), MIPI (prognostic score for mantle cell lymphoma), and PIT (prognostic index for peripheral T cell lymphoma) or by conventional computed tomography (CT)–based response assessment [5–9]. Therefore, there is an urgent need for new prognostic and predictive markers which permit an accurate and early identify high-risk patient categories.

^[18]^Fluorine fluorodeoxyglucose-positron emission tomography/computed tomography (FDG-PET/CT) has been recognized by the 2014 International Conference on Malignant Lymphoma imaging consensus guidelines as the standard imaging modality to evaluate glucose metabolism in fluorodeoxyglucose (FDG)-avid lymphoma tumors [10,11]. Its value for prognosis prediction at interim and end treatment has been recently investigated [12–14]. Many studies have also showed that quantitative volumetric parameters derived from baseline ^18^F-FDG PET such as total metabolic tumor volume (TMTV) or total lesion glycolysis (TLG) could predict outcome in diffuse large B-cell lymphoma [15–17], in follicular lymphoma [18], in peripheral T-cell lymphoma [19], in extranodal natural killer/T-cell lymphoma [20] and in Hodgkin lymphoma [21,22]. However, those studies evaluating the prognostic values of pre-therapy TMTV and TLG in in patients with various lymphoma subtypes showed inconclusive and contradictory results [23].

Therefore, the purpose of this meta-analysis was to evaluate the prognostic value of baseline TMTV or TLG by PET/CTin patients with lymphoma, in order to provide more evidence of their clinical value as prognostic biomarkers.

## Materials and methods

### Inclusion and exclusion criteria

A computerized search of PubMed, Embase and Cochrane was conducted to find relevant studies published prior to May 01, 2018. The following search terms were used: ("lymphoma"[MeSH Terms] OR lymphom*[All Fields] OR lymphoproliferative [All Fields] OR hodgkin*[All Fields] OR non-hodgkin* [All Fields]) AND ("Tomography, emission-computed"[MeSH Terms] OR ("positron emission tomograpy"[MeSH Terms]) OR (computed [All Fields] AND tomograph*[All Fields])) AND (prognos* OR predict* OR surviv* OR overall survival* OR recurrence* OR progress*). All searches were limited to human studies.

Eligible studies met the following criteria: (i) They were observational studies (retrospective or prospective) or clinical trials, (ii) the studies were limited to lymphoma, (iii) ^18^F-FDG PET was used as an initial imaging tool, (iv) patients had not undergone chemotherapy, immune-chemotherapy or radiotherapy before the ^18^F-FDG PET scan, (v) the volume of the lymphoma was measured, (vi) the survival data was reported. Studies were excluded if: (i) they were case reports, case series, review articles, editorials, letters or comments; (ii) the patient survival data was unavailable or insufficient to perform the meta-analysis, (iii) the data included was specifically for HIV-associated lymphoma, pediatric lymphoma, primary central nervous system lymphoma, primary testicular lymphoma, or primary mediastinal large B-cell lymphoma, or (iv) they included overlapping patients and data. Two reviewers (B.P. Guo and Q. Ke) independently selected the literature using a standardized protocol. Disagreements were resolved by discussion.

### Quality assessment

The methodological quality of the primary manuscripts was independently evaluated by two reviewers (B.P. Guo and X.H. Tan) by means of the Newcastle-Ottawa-Scale (NOS) [24], which is used for the quality assessment of cohort and case-control studies. The NOS comprises three quality parameters: selection (0–4 points), comparability (0–2 points), and outcome assessment (0–3 points). Studies with scores of six or more were considered to be of high quality. Any disparities between investigators were resolved by discussion. Study quality was not an exclusion criterion.

### Data extraction

Data extraction was carried out by B.P Guo and independently confirmed by the other authors (X.H. Tan and H. Cen). The collected data included the following: Study characteristics: first author, year of publication, country, study design, imaging modalities, type of lymphoma, number of patients, treatment, tumor volume parameters (maximum threshold for PET volume auto-segmentation, and median MTV/TLG), MTV/TLG cut-off values, determination of MTV cut-off, median follow-up, and endpoints. Extracted data were entered onto a standardized Excel file (Microsoft Corporation). Discrepancies were resolved by discussion with coauthors.

We chose OS and PFS as endpoints for our meta-analysis. Overall survival is defined as the length of time from either the date of diagnosis or the date of recruitment in a study to the moment of death as a result of any cause. PFS is defined as the length of time from either the date of diagnosis or the date of recruitment in a study until lymphoma progression or death as a result of any cause.

### Statistical analysis

The impact of MTV or TLG on survival was measured by estimating the effect size of the hazard ratios (HR). Pooled HRs of more than 1.00 indicated poor survival in the group with high MTV or TLG values when compared with the group with low values. For studies in which the HRs and CIs were not available, we used the method proposed by Parmar et al. [25] to derive estimates from survival curves. The point estimate of the HR was considered statistically significant at the *p*< 0.05 level if the 95% CI did not include the value 1.

Heterogeneity was assessed by means of Cochran *Q* and *I*^*2*^ statistics. I-square (*I*^*2*^) values of <30%, 30%-50%, 50%-75% and >75% were defined as low, moderate, substantial and considerable heterogeneity, respectively [26]. If heterogeneity existed between primary studies, a random effects model was used. Otherwise, a fixed effects model was used for the meta-analysis. Publication bias was assessed by visual inspection of the funnel plot and also by means of the Begg and Egger tests [27,28]. A p-value less than 0.05 indicates the existence of publication bias. All statistical analyses were performed by using RevMan 5.3 (Nordic Cochrane Centre).

## Results

### Literature search procedure

**Fig 1:**
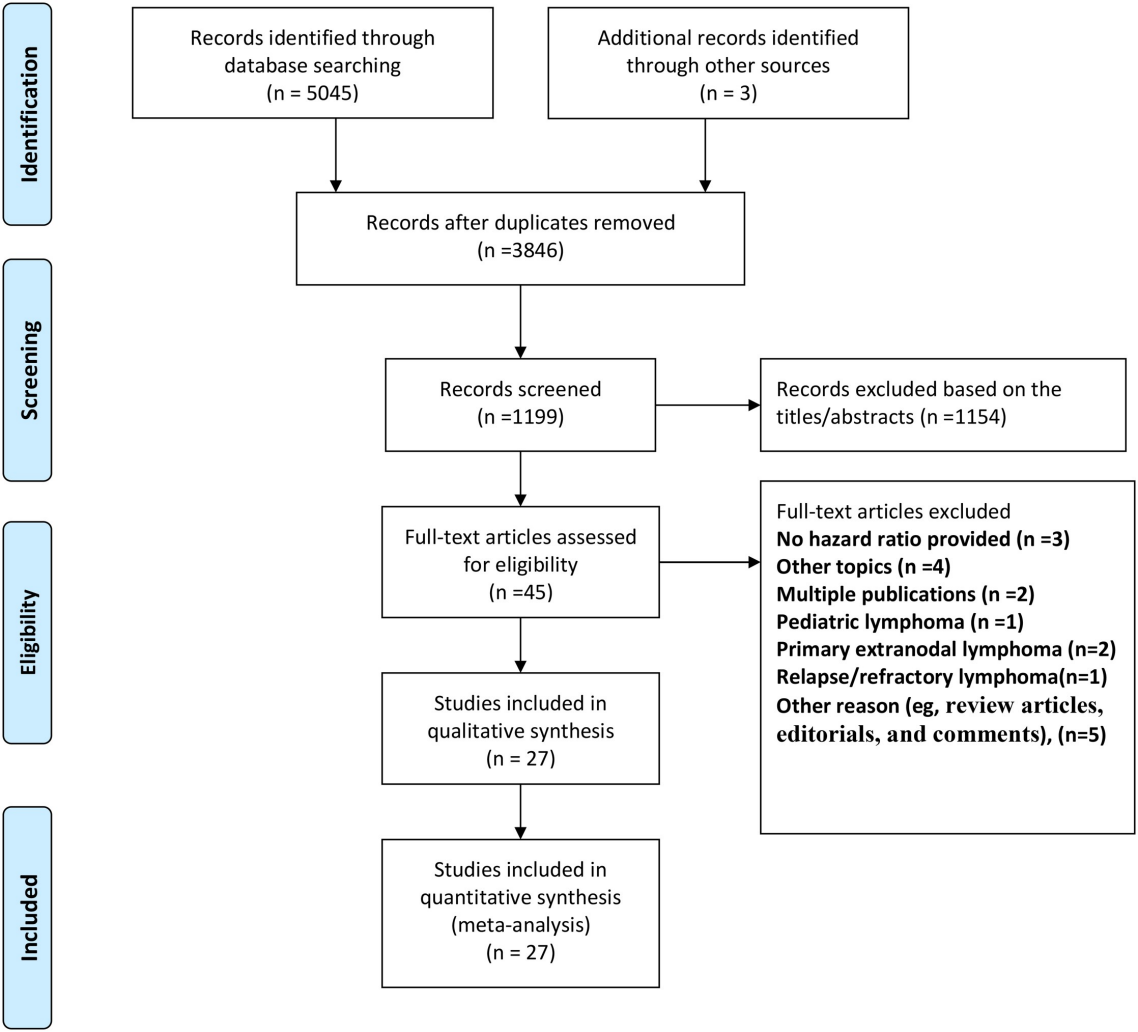
Flow diagram of the systematic review and meta-analysis process.

The PRISMA statement flowchart shows the process of literature screening and selection, as well as the reasons for exclusion (Fig 1). Our initial search yielded 5045 articles. After removing duplicates and screening the titles and abstracts, 45 articles were reviewed in more detail. Of the 45 full-text studies, 18 were excluded for the following reasons: 7 studies had incomplete or unavailable data and other topics; 5 reports were reviews and editorials; 1 study involved pediatric lymphoma; 3 studies included primary extranodal lymphoma and relapsed or refractory lymphoma; 2 studies were multiple publications. After reviewing the full texts, 27 studies were finally selected as potentially appropriate for inclusion in the meta-analysis [15–22,29–47].

### Study characteristics

The 27 observational studies fulfilled the inclusion criteria, were published between 2012 and 2018 and are summarized in Table 1. Twenty-five studies were retrospective observational studies, and three studies were prospective multicenter trials. Seventeen studies included patients with diffuse large B cell lymphoma, three included patients with follicular lymphoma, one included patients with peripheral T-cell lymphoma, four studies included patients with extranodal natural killer/T cell lymphoma, and three studies included patients with Hodgkin lymphoma. The total sample size was 2729. Either TMTV or TLG was measured in 12 studies, and both were measured in 15 studies.

**Table 1:** Characteristics of studies included in the meta-analysis.

Study	Year	Country	Study Design	NOS	Type of Lymphoma	Patients (No.)	Treatment regimen	Tumor Volume Parameters(MTV/TLG)			Cut-off values		Determination of MTV Cut off	Endpoints	Median follow-up
								Threshold (%)	Median MTV (cm^3^)	Median TLG	MTV (cm^3^)	TLG			
Song et al.^a^	2012	Korea	R	8	DLBCL	169	R-CHOP	≥2.5	198.1	NR	220	NR	ROC analysis	PFS/OS	36 months
Manohar et al.	2012	India	R	6	NHL^§^	51	R-CHOP-like	Background- level ^†^	957	5356	416	3340	ROC analysis	PFS/OS	12 months
Kim et al.	2013	Korea	R	8	DLBCL	140	R-CHOP	Various^‡^	NR	415.5	NR	415.5	N/A	PFS/OS	28.5 months
Oh et al.	2013	Korea	R	6	DLBCL	181	R-CHOP	≥2.5	156.89	NR	65.975	NR	ROC analysis	PFS/OS	NR
Song et al.	2013	Korea	R	7	HL	127	ABVD	≥2.5	142.6	NR	198	NR	ROC analysis	PFS/OS	45.8 months
Kim et al.	2013	Korea	R	5	ENKTL	20	CTx follow RT/ Only CTx	NR	10.7	46.9	14.4	52.7	ROC analysis	PFS/OS	26.3 months
Esfahani et al.	2013	USA	R	7	DLBCL	20	R-CHOP	50	379.16	704.77	379.16	704.77	ROC analysis	PFS	mean 51.35 months
Sasanelli et al.	2014	France	R	9	DLBCL	114	R-CHOP/ R-ACVBP	41	315	2974	550	4,576	ROC analysis	PFS/OS	39 months
Gallicchio et al.	2014	Italy	R	5	DLBCL	52	R-CHOP-like	42	43	596.9	16.1	589.5	ROC analysis	EFS/OS	18 months
Kim et al.	2014	Korea	R	5	DLBCL	96	R-CHOP	≥2.5	130.7	NR	130.7	NR	ROC analysis	EFS/OS	27.8 months
Adams et al.	2015	Netherland	R	9	DLBCL	73	R-CHOP	40	272	2955.4	272	2955.4	ROC analysis	PFS/OS	2.7years
Schoder et al.	2015	USA	P	6	DLBCL	65	R-CHOP	Various^‡^	226	NR	NR	NR	N/A	PFS/OS	51 months
Kanoun et al.	2015	France	R	8	HL	59	CTx ± RT	Various^‡^	160	NR	313	NR	ROC analysis	PFS/OS	39 months
Mikhaeel et al.	2016	UK	R	8	DLBCL	147	R-CHOP	41	595	4669.5	396	4541	ROC analysis	PFS	3.8 years
Zhou et al.	2016	China	R	8	DLBCL	91	R-CHOP	Background-level ^†^	50.7	497.3	PFS: 70 OS: 78	PFS: 827 OS: 726	ROC analysis	PFS/OS	30 months
Song et al.^b^	2016	Korea	R	6	DLBCL	107	R-CHOP	≥2.5	526.8	NR	601.2	NR	ROC analysis	PFS/OS	40.8 months
Cottereau et al.	2016	France	R	8	PTCL	108	CHOP-like/ ACVBP	41	224	1155	230	1068	ROC analysis	PFS/OS	23 months
Meignan et al.	2016	France	R	9	FL 1-3a	185	R-CHOP/ R-CVP/R-FM	41	297	NR	510	NR	X-tile analysis	PFS/OS	64 months
Chang et al.	2017	China	R	6	ENKTL	52	DDGP/SMILE	40	11.2	46.4	16.1	44.7	ROC analysis	PFS/OS	19 months
Chang et al.	2017	Taiwan	R	7	DLBCL	118	R-CHOP	≥2.5	550.4*	3533.2*	165.4	1204.9	ROC analysis	PFS/OS	28.7 months
Kesavan et al.	2017	Australia	P	7	FL	68	Iodine-131-rituximab	41	510	NR	510	NR	NR	TTNT/OS	59 months
Song et al.	2017	Korea	R	5	ENKTL	100	CTx follow RT/CTx	≥2.5	36.2	NR	94.2	NR	ROC analysis	PFS/OS	NR
Cottereau et al.	2018	France	P	9	HL	258	ABVD	41	67	332	147	495	X-tile and ROC analysis	PFS/OS	55 months
Ding et al.	2018	China	R	7	DLBCL	72	R-CHOP	40	139.48	1413.77	67.71	1413.77	ROC analysis	PFS/OS	45 months
Toledano et al.	2018	France	R	8	DLBCL	114	R-CHOP/ R-CHOP-like	41	275.8	NR	261.4	1325.80	ROC analysis	PFS/OS	40 months
Delfau-Larue et al.	2018	France	R	8	FL	133	R-CHOP/ R-CHOP-like/Chemo-free	41	354	NR	510	NR	X-tile and ROC analysis	PFS/OS	48 months
Pak et al.	2018	Korea	R	7	ENKTL	36	NR	40	NR	NR	7	45.8	NR	RFS/OS	20.6 months

PET/CT, positron emission tomography/computed tomography; MTV, metabolic tumor volume; TLG, total lesion glycolysis; NOS, the Newcastle-Ottawa-Scale; DLBCL, diffuse large B cell lymphoma; HL, hodgkin lymphoma; NHL, non-hodgkin’s lymphoma; ENKTL, extranodal natural killer/T cell lymphoma; PTCL, peripheral T-cell lymphoma; FL, follicular Lymphoma; R, retrospective; P, prospective; NR, not reported; PFS, progression-free surviva; TTNT:time-to-next-treatment; IQR, interquartile range; ROC, receiver operator curve; N/A, not applicable; R-CHOP: rituximab, cyclophosphamide, doxorubicin, vincristine, and prednisone; ABVD, doxorubicin,bleomycin, vinblastine, dacarbazine; CTx, chemotherapy; RT, radiotherapy; R-ACVBP: rituximab, doxorubicine, vindesine, cyclophosphamide, bleomycin, prednisolone; R-ICE, rituximab, etoposide, ifosfamide, carboplatin; R-CVP, rituximab, cyclophosphamide, vincristine and prednisolone; R-FM, rituximab, fludarabine and mitoxantrone; DDGP, dexamethasone, cisplatin, gemcitabline and pegaspargase; SMILE, dexamethasone, methotrexate, ifosfamide, L-asparaginase and etoposide;

^‡^ tested various proposed thresholds, including 41%;

^†^ MTV was measured by setting the tumor marginal threshold of liver SUVmean plus 3SDs. SUVmean in liver was calculated in a standard-sized ROI of 3cm in diameter;

^§^ Of the 51 patients, 39 (77%) had DLBCL, 8 had anaplastic large T-cell lymphoma and 4 had high-grade peripheral T-cell lymphoma.

^a^, In staged II and III patients without extranodal site involvement; b, In patients with bone marrow involvement of lymphoma.

*The mean values of total MTV and TLG were 550.4 ± 678.3 cm3 and 3533.2 ± 4394.1 cm3 respectively.

Three thresholding methods for the auto segmentation of PET volumes exist. A fixed SUV of 2.5 was used in 7 studies, the percentage of SUVmax (40%, 41%, 42% or 50%) was used in 18 studies, and in 2 studies the MTV was measured by setting the tumor margin threshold as the liver SUVmean plus 3SDs. In each study, patients were divided into 2 groups (high and low volume) based on the cut-off values. The MTV and TLG cut-off values were determined by means of receiver operating curve (ROC) and X-tile analyses. Receiver operating characteristics (ROCs) was used in 19 studies, receiver-operating characteristics and X-tile analysis in 3 studies, X-tile analysis in 1 study, and 4 studies did not provide cut-off information. The MTV cut-off values ranged between 10.7 and 595 cm^3^ and the TLG values ranged from 46.4 to 5356. The study quality assessed by means of the NOS was fair, with a median quality score of 8 (range 5–9).

### Prognostic value of baseline metabolic tumor volume

**Fig 2:**
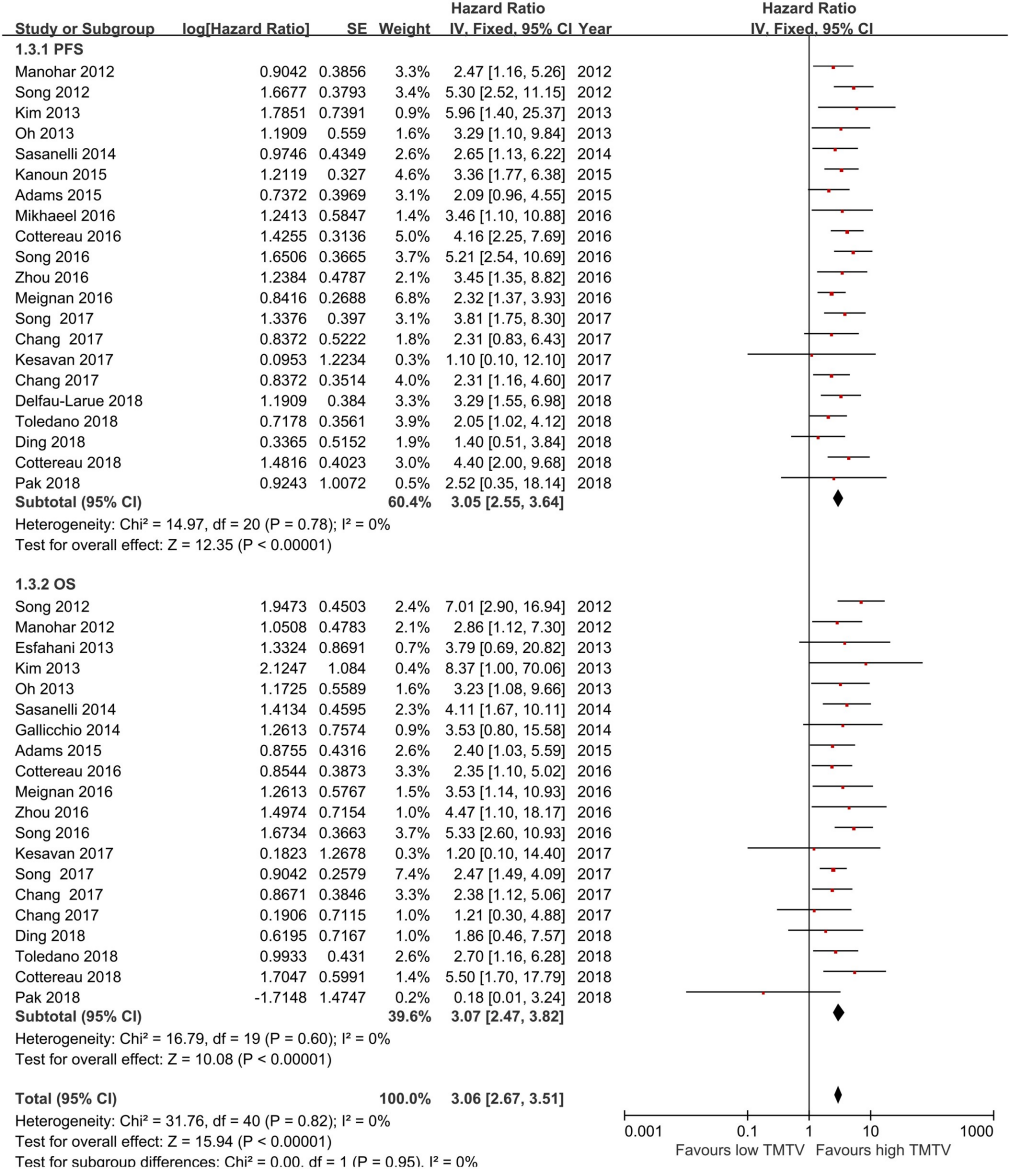
Meta-analysis of the hazard ratios for PFS, and OS for high TMTV vs low TMTV. Hazard ratios and 95% confidence intervals from individual studies are depicted as squares and horizontal lines, respectively. The pooled estimate is shown as a diamond shape, where the center represents the pooled HR and the horizontal borders represent the 95% CI. Hazard ratios are defined as high TMTV vs low TMTV, therefore a hazard ratio >1 represents a higher risk of death or progression associated with high TMTV. TMTV = total metabolic tumor volume, OS = overall survival, PFS = progression-free survival, CI = confidence interval.

Twenty-one studies [15–19,21,22,29,30–35,37,39,43–47] on adult lymphoma were included for the analysis of the relationship between TMTV and PFS. The results of our meta-analysis showed that a high TMTV was associated with a shorter PFS than a low TMTV, with a pooled HR of 3.05 (95% CI, 2.55–3.64, *p*<0.00001). No significant heterogeneity was found across studies (*I*^*2*^ = 0.0%, *P* = 0.78). Twenty-one studies [15,17–20,22,29–35,38–40,43–47] reported data on TMTV and OS in adult lymphoma. The meta-analysis results demonstrated that a high TMTV was associated with shorter OS than a low TMTV, with a pooled HR of 3.07 (95%CI, 2.47–3.82, *p*<0.00001). No significant heterogeneity was found across studies (*I*^*2*^ = 0.0%, *p* = 0.60; Fig 2).

Next, we examined the relationship between TMTV and the clinical outcome of different types of lymphomas. A meta-analysis of thirteen studies of DLBCL patients showed poorer PFS and OS in patients with high TMTV than in those with low TMTV, with pooled HRs for PFS and OS of 2.93 (95%CI, 2.29–3.73, *p*<0.001; heterogeneity: *I*^*2*^ = 0.0%, *p* = 0.481) and 3.52 (95%CI, 2.67–4.64, *p*<0.001; heterogeneity: *I*^*2*^ = 0.0%, *p* = 0.806), respectively. Three studies examined the prognostic significance of high TMTV in FL patients, and the pooled HRs for PFS and OS were 2.55 (95%CI, 1.65–3.92, *p*<0.001; heterogeneity: *I*^*2*^ = 0.0%, *p* = 0.622) and 2.89 (95%CI, 1.04–7.99, *p*<0.001; heterogeneity: *I*^*2*^ = 0.0%, *p* = 0.419), respectively. Four studies assessed the prognostic significance of high TMTV in ENKL patients, and the pooled HRs for PFS and OS were 3.25 (95%CI, 1.75–6.07, *p*<0.001; heterogeneity: *I*^*2*^ = 10.7%, *p* = 0.340) and 2.24 (95%CI, 1.23–4.08, *p* = 0.008; heterogeneity: *I*^*2*^ = 24%, *p* = 0.267), respectively. Two studies investigated the prognostic significance of high TMTV in HL patients, and the pooled HR for PFS was 3.89 (95%CI, 2.19–6.90, *p*<0.001; heterogeneity: *I*^*2*^ = 0.0%, *p* = 0.646) (Table 2). Notably, only one study provided relevant data on the correlation between TMTV and PTCL outcome and only one study provided data on the correlation between TMTV and OS in HL patients; therefore, the pooled analysis could not be performed.

**Table 2:** Pooled hazard ratios for PFS and OS according to TMTV.

Study selection	N	PFS				N of cohorts	OS			
		Random-effects model		Heterogeneity			Random-effects model		Heterogeneity	
		Pooled HR (95% CI)	P value	I^*2*^ (%)	P_*H*_ value		Pooled HR (95% CI)	P value	I^*2*^ (%)	P_*H*_ value
**Type of Lymphoma**										
DLBCL	12	2.93(2.29–3.73)	<0.001	0	0.481	13	3.52(2.67–4.64)	<0.001	0	0.806
FL	3	2.55(1.65–3.92)	<0.001	0	0.622	2	2.89(1.04–7.99)	<0.001	0	0.419
PTCL	1	4.16(2.25–7.68)	<0.0001	-	-	1	2.35(1.10–5.04)	0.028	-	-
ENKTL	4	3.25(1.75–6.07)	<0.001	10.7	0.34	4	2.24(1.23–4.08)	0.008	24	0.267
HL	2	3.89(2.19–6.90)	<0.001	0	0.646	1	3.90(1.60–9.50)	0.0032	-	-
**Study Design**										
Retrospective	19	3.20(2.64–4.01)	<0.001	0	0.774	17	3.21(2.54–4.07)	<0.001	0	0.743
Prospective	3	2.81(1.72–4.59)	<0.001	11.2	0.342	3	1.91(0.51–7.15)	0.337	23.1	0.273
**Sample size**										
≥100	11	3.51(2.78–4.42)	<0.001	0	0.667	10	3.59(2.70–4.76)	<0.001	0	0.531
<100	7	2.82(1.99–3.99)	<0.001	0	0.55	9	2.45(1.65–3.65)	<0.001	0	0.809
**Threshold**										
≥2.5	5	3.93(2.76–5.60)	<0.001	0	0.553	5	3.65(2.38–5.61)	<0.001	28.9	0.229
41	6	2.56(1.83–3.58)	<0.001	0	0.554	5	3.56(2.22–5.70)	<0.001	0	0.772
40	3	2.19(1.21–3.98)	0.01	0	0.98	3	1.80(0.84–3.83)	0.129	5	0.349
other	4	3.20(2.02–5.05)	<0.001	0	0.753	4	3.69(1.89–7.22)	<0.001	0	0.817

N: number of studies; HR: hazard ratio; 95% CI: 95% confidence interval; P_*H*_: p values of Q test for heterogeneity test; OS: Overall survival; PFS: Progression free survival.

We also conducted subgroup analyses stratified by data collection method, sample size, and different threshold values. The subgroup analysis of retrospectively collected data showed pooled HRs for PFS and OS of 3.20 (95%CI, 2.64–4.01, *p*<0.001; heterogeneity: *I*^*2*^ = 0.0%, *p* = 0.774) and 3.21 (95%CI, 2.54–4.07, *p*<0.001; heterogeneity: *I*^*2*^ = 0.0%, *p* = 0.743), respectively. The prospectively collected data showed pooled HRs for OS and PFS of 2.81 (95%CI, 1.72–4.59, *p*<0.001; heterogeneity: *I*^*2*^ = 11.2%, *p* = 0.342) and 1.91 (95%CI, 0.51–7.15, *p* = 0.337; heterogeneity: *I*^*2*^ = 23.1%, *p* = 0.273), respectively. The subgroup analysis performed on the basis of sample size showed that the negative predictive value of high TMTV on PFS and OS was present both in samples with sizes ≥100 (PFS, HR: 3.51, 95%CI: 2.78–4.42, *p*<0.001; OS, HR: 3.59, 95%CI: 2.70–4.76, *p*<0.001) and <100 (PFS, HR: 2.82, 95%CI: 1.99–3.99, *p*< 0.001; OS, HR: 2.45, 95%CI:1.65–3.65, *p*<0.001). Lastly, the subgroup analyses by thresholds (≥ 2.5, 40%, 41%, and others) demonstrated that a high TMTV was associated with shorter PFS and OS than a low TMTV (Table 2).

### Prognostic value of baseline total lesion glycolysis

**Fig 3:**
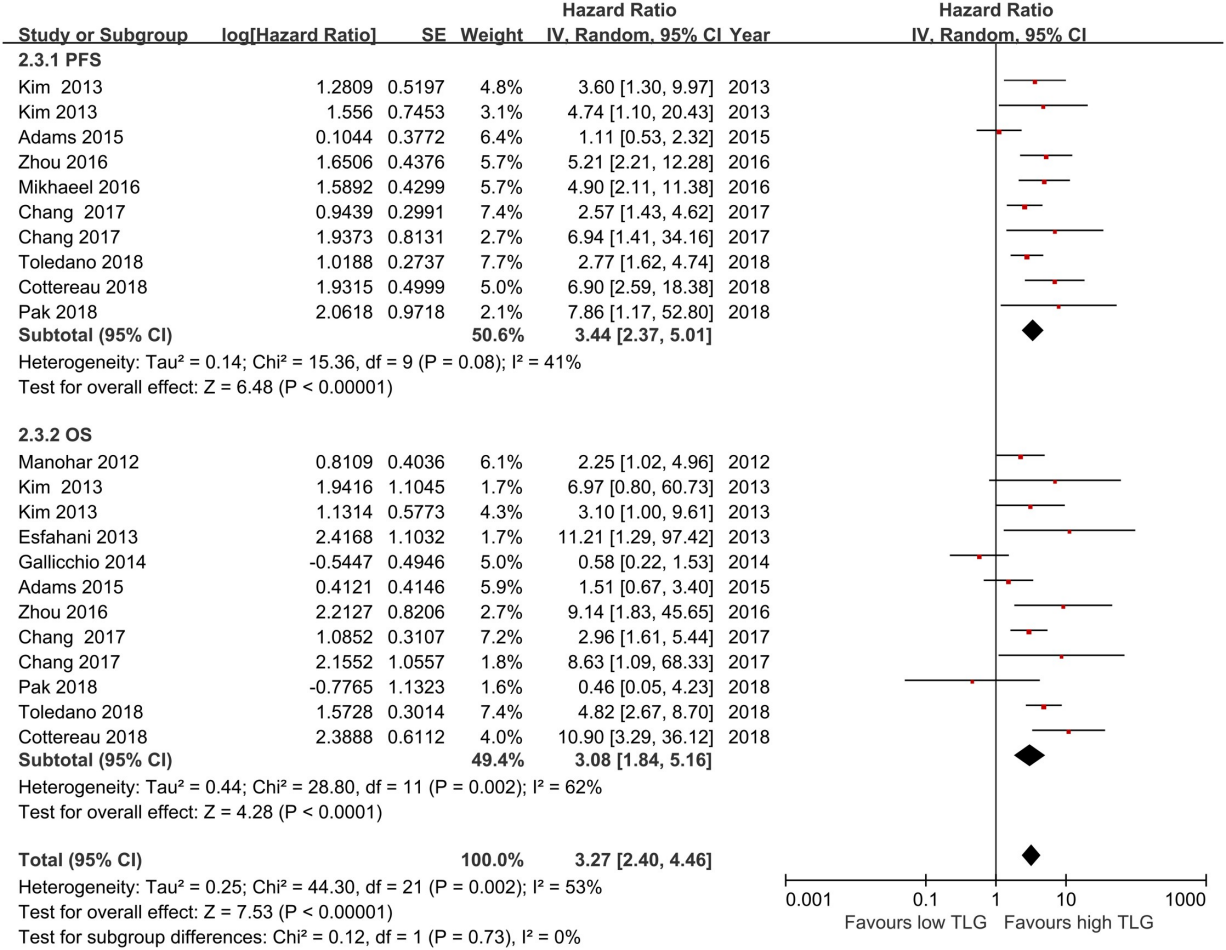
Meta-analysis of the hazard ratios for PFS and OS for high TLG vs low TLG. Hazard ratios and 95% confidence intervals for death or progression associated with high vs low TLG. TLG = total lesion glycolysis, OS = overall survival, PFS = progression-free survival, CI = confidence interval.

Ten studies [15–17,20,22,29,32,37,43,46] were included for the analysis of the relationship between TLG and PFS in adult lymphoma. The results of our meta-analysis showed that high TLG values were associated with shorter PFS than low TLG values, with a pooled HR of 3.44 (95%CI, 2.37–5.01, *p*<0.00001). There was moderate heterogeneity across the studies, but it did not reach statistical significance (*I*^*2*^ = 41.0%, *p* = 0.08). Twelve studies [15,17,20,22,29,32,34,37,38,40,43,46] reported data on TLG and OS in adult lymphoma. Because significant heterogeneity was found across the studies (*I*^*2*^ = 62.0%, *P* = 0.002), a pooled HR of 3.08 (95%CI, 1.84–5.16, *p =* 0.0001) was calculated on the basis of a random-effects model (Fig 3).

Next, we examined the prognostic significance of TLG on different types of lymphomas. A meta-analysis of seven and eight studies involving DLBCL patients showed poorer PFS and OS in those with high TMTV values than in those with low TMTV values, with pooled HRs for OS and PFS of 3.06 (95%CI, 1.52–6.18, *p* = 0.002; heterogeneity: *I*^*2*^ = 67.3%, *p* = 0.003) and 2.93 (95%CI, 1.89–4.53, *p*<0.001; heterogeneity: *I*^*2*^ = 49.5%, *p* = 0.065), respectively. Three studies examined the prognostic significance of high TLG values in ENKL patients, and the pooled HRs for PFS and OS were 2.99 (95%CI, 1.83–4.89, *p*<0.001; heterogeneity: *I*^*2*^ = 0.0%, *p* = 0.503) and 2.58 (95%CI, 1.33–5.01, *p* = 0.005; heterogeneity: *I*^*2*^ = 19.0%, *p* = 0.291), respectively (Table 3). Only one study provided relevant data on the correlation between TLG and clinical outcome in PTCL patients and only one study provided data on the correlation between TLG and outcome in HL patients; therefore, the pooled analysis could not be performed.

**Table 3:** Pooled hazard ratios for PFS and OS according to TLG.

Study selection	N	PFS				N	OS			
		Random-effects model		Heterogeneity			Random-effects model		Heterogeneity	
		Pooled HR (95% CI)	P value	I^*2*^ (%)	P_*H*_ value		Pooled HR (95% CI)	P value	I^*2*^ (%)	P_*H*_ value
**Type of Lymphoma**										
DLBCL	7	2.93(1.89–4.53)	<0.001	49.5	0.065	8	3.06(1.52–6.18)	0.002	67.3	0.003
PTCL	-	-	-	-	-	-	-	-	-	-
ENKTL	3	2.99(1.83–4.89)	<0.001	0	0.503	3	2.58(1.33–5.01)	0.005	19	0.291
HL	1	6.90(2.59–18.36)	0.0001	-	-	1	10.9(3.29–36.23)	0.0001	-	-
**Study Design**										
Retrospective	10	2.97(2.03–4.35)	<0.001	35.7	0.122	11	2.28(1.40–3.71)	0.001	50.1	0.029
Prospective	1	6.90(2.59–18.36)	0.0001	-	-	1	10.9(3.29–36.23)	0.0001	-	-
**Sample size**										
≥100	5	4.60(2.83–7.47)	<0.001	0	0.59	4	3.54(1.56–8.06)	0.003	62.6	0.045
<100	6	2.73(1.53–4.86)	0.001	48.7	0.083	9	2.07(1.21–3.55)	0.008	54.9	0.023
**Threshold**										
≥2.5	1	6.94(1.41–34.12)	0.017	-	-	1	8.63(1.09–68.34)	0.041	-	-
41	3	4.64(2.44–8.85)	<0.001	13.9	0.313	1	10.9(3.29–36.23)	0.0001	-	-
40	3	2.16(0.94–4.96)	0.07	60.6	0.079	3	1.90(0.91–3.98)	0.086	43.7	0.169
other	3	4.52(2.48–8.21)	<0.001	0	0.86	5	3.53(1.98–6.28)	<0.001	3.1	0.389

N: number of studies; HR: hazard ratio; 95% CI: 95% confidence interval; P_*H*_: p values of Q test for heterogeneity test; OS: Overall survival; PFS: Progression free survival.

We also conducted subgroup analyses stratified by data collection method, sample size, and different threshold values. The subgroup analysis of retrospectively collected data showed pooled HRs for OS and PFS of 2.28 (95%CI, 1.40–3.71, *p* = 0.001; heterogeneity: *I*^*2*^ = 50.1%, *p* = 0.029) and 2.97 (95%CI, 2.03–4.35, *p*<0.001; heterogeneity: *I*^*2*^ = 35.7%, *p* = 0.122), respectively. Only one study involved prospectively collected data, so the pooled analysis could not be performed. The subgroup analysis conducted by sample size showed that the negative predictive value of high TMTV on PFS and OS was present both in samples with sizes ≥100 (PFS, HR: 4.60, 95%CI: 2.83–7.47, *p*<0.001; OS, HR: 3.54, 95%CI: 1.56–8.06, *p*<0.001) and <100 (PFS, HR: 2.73, 95%CI: 1.53–4.86, *p =* 0.001; OS, HR: 2.07, 95%CI:1.21–3.55, *p =* 0.008). A meta-analysis of three studies with a 40% threshold showed there was a trend for a correlation between high TLG values and lymphoma prognosis, with pooled HRs for OS and PFS of 1.90 (95%CI, 0.91–3.98, *p* = 0.086; heterogeneity: *I*^*2*^ = 43.7%, *p* = 0.169) and 2.16 (95%CI, 0.94–4.96, *p* = 0.07; heterogeneity: *I*^*2*^ = 60.6%, *p* = 0.079), respectively. The meta-analysis of studies using other thresholds (including the liver SUVmean plus 3SDs, 50%) showed a negative correlation between high TLG values and lymphoma prognosis, with pooled HRs for OS and PFS of 3.53 (95%CI, 1.98–6.28, *p*<0.001; heterogeneity: *I*^*2*^ = 3.1%, *p* = 0.389) and 4.52 (95%CI, 2.48–8.21, *p*<0.001; heterogeneity: *I*^*2*^ = 0.0%, *p* = 0.860), respectively. The subgroup analysis selecting a 41% threshold showed a negative predictive value of high TLG on PFS, with a pooled HR for PFS of 4.64 (95%CI, 2.44–8.85, *p*<0.001; heterogeneity: *I*^*2*^ = 13.9%, *p* = 0.313) (Table 3). Only one study used a threshold ≥2.5, so the pooled analysis could not be performed.

### Publication bias

**Fig 4:**
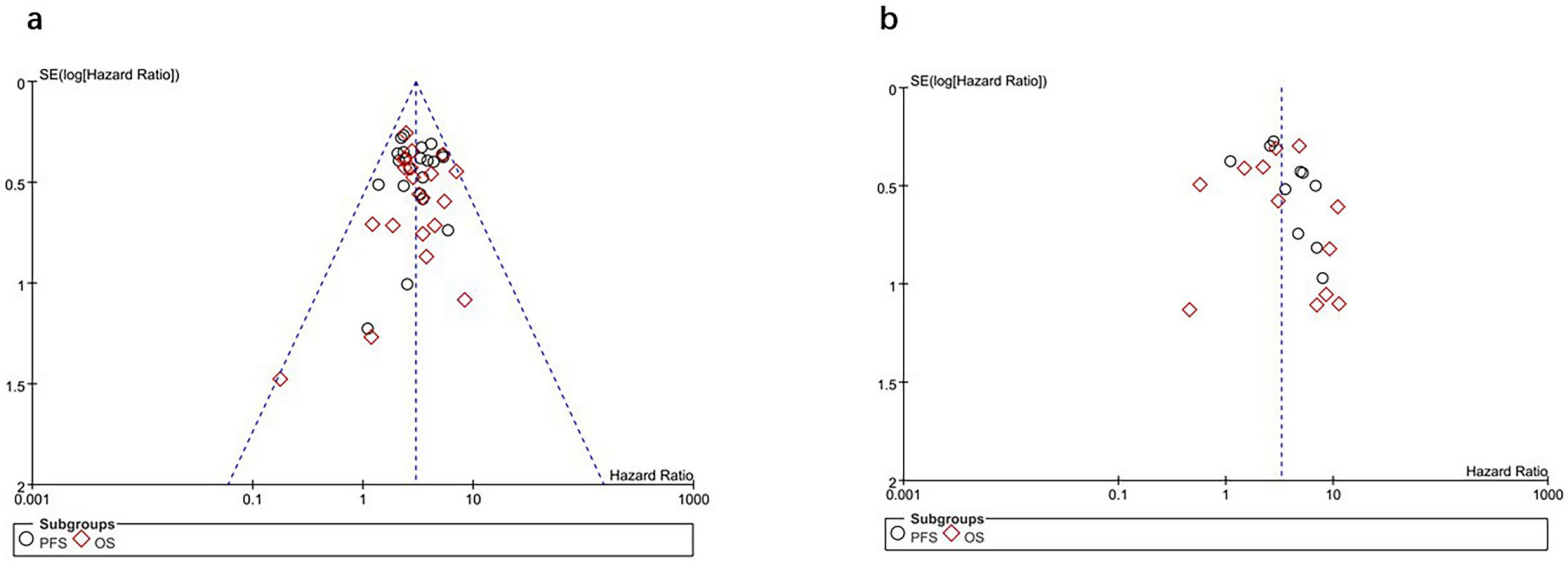
Assessment of publication bias using Funnel plot analysis. (a) Funnel plot of hazard ratio for overall survival and progression-free survival for high TMTV (horizontal axis) and the standard error (SE) for the hazard ratio (vertical axis). (b) Funnel plot of hazard ratio for overall survival and progression-free survival for high TLG (horizontal axis) and the standard error (SE) for the hazard ratio (vertical axis). Each study is represented by one circle. The vertical line represents the pooled effect estimate.

Inspection of the funnel plot and formal statistical tests (**TMTV:** Egger test, ***p*** = **0**.**931**; Begg test, ***p*** = **0.867**; **TLG:** Egger test, ***p*** = **0.200**; Begg test, ***p*** = **0.236**; Fig 4) showed no evidence of publication bias in the meta-analysis of the prognostic significance of baseline metabolic tumor volume and total lesion glycolysis in adult lymphoma.

## Discussion

### Main findings

This meta-analysis comprehensively and systematically reviewed the current available literature and found that: (1) A high baseline TMTV significantly predicted poor OS and shorter PFS in adult lymphoma patients (p<0.00001 and p<0.00001, respectively); (2) A high baseline TMTV was significantly associated with reduced survival in DLBCL patients treated with R-CHOP and predicted poor OS and PFS for different types of lymphomas, such as FL, ENKL and HL. The evidence supporting this association was consistent in most subgroup analyses (retrospective data collection, ethnicity, sample size, and different thresholds). The analysis of prospectively collected data and studies using a 40% threshold suggested a trend towards poor OS; however, these results were not statistically significant; (3) A high baseline TLG significantly predicted poor OS and shorter PFS in adult lymphoma patients (*p*<0.00001 and *p* = 0.005, respectively); (4) A high baseline TLG was significantly associated with reduced survival in DLBCL patients treated with R-CHOP and predicted poor OS and PFS in different types of lymphomas, such as FL, ENKL and HL. The evidence of this association was consistent in most subgroup analyses (data collection method, ethnicity, sample size, and different thresholds).

Metabolic tumor volumes can be segmented by using various methods, such as a fixed SUV threshold, a percentage (based on the percentage of maximum uptake in the lesion), a threshold adjusted to the tumor-to-background ratio, or a gradient [23]. Reproducibility is the key for reliable volumetric tumor segmentation. Different TMTV measurement methods have been used in various types of lymphoma, each with specific advantages and disadvantages. A method based on the 41% SUVmax threshold is recommended by the European Association of Nuclear Medicine (EANM) for TMTV measurement of solid tumors. It has been developed in patients with HL and DLBCL, showing good reproducibility [48]. Different thresholding methods were used for PET volume auto-segmentation in the studies included herein; however, a threshold of 41% or 40% of the SUVmax was widely used. In our study, we conducted subgroup analyses stratified by different thresholds (≥2.5, 41%, and others). The results demonstrated that high TMTV or TLG values were associated with shorter PFS and OS. Subgroup stratification based on a threshold of 40% of the SUVmax showed that a high TMTV was a negative predictor of PFS; however, it did not significantly predict poor PFS and OS in the case of TLG.

Baseline MTV by PET/CT, is a promising prognostic indicator in patients with lymphoma, which is better than using size-defined bulk [16, 33]. TLG, which is the MTV multiplied by the mean SUV in the volume, is also prognostic [37], but appears no better than MTV in prediction of survival in lymphoma [16].

Several retrospective studies have shown that metabolic tumor volume (MTV) is a strong predictor of prognosis irrespective of the method [19,21,49]. However, cut-offs used to divide patients into high and low risk groups by MTV are highly dependent on the patient population and the method used. A fixed 41% SUVmax relative thresholding method has been applied successfully in different subtypes of lymphoma, but probably overestimated the volume of lesions with low SUVmax, particularly for smaller VOIs [19,21,48]. The 2.5 method could include the volume of nontumor regions located between small distant nodes with high uptake [50]. The 2.5 method probably overestimated MTV in approximately 12% of patients who had low FDG uptake in the liver or liver involvement by lymphoma [49]. Furthermore, the negative and positive predictive values of the 41% method have been shown to be superior to other methods, which results in excellent outcome prediction in other subtypes of lymphoma [21]. Generally, current evidence showed that metabolic tumor volume values were significantly influenced by the choice of the method used for determination of volume. However, no significant differences were found in term of prognosis [21]. In clinical practice, a consensus on the most accurate method or an optimal cut-off to define the MTV for specific lymphoma subtypes will be required, which will require validation in multicenter prospective trials.

Several methods for autosegmentation of PET volumes exist (e g, threshold-based, gradient-based, statistical, and texture-based methods) [51]. All methods have strengths and limitations. Reproducibility is the key for tumor segmentation in routine practice [23]. There is no universally accepted reproducible and practical method for tumor segmentation. Recently, Yu et al. reported a new semi-automatic approach that applies first an anatomical multi-atlas segmentation on the CT images to remove the organs having hyper uptake value on PET images. Using a CRFs (Conditional Random Fields) model, the rate of good detection of lymphoma is 100% in 11 patients [52]. Meanwhile, this new semi-automatic approach has the best dice index for the real lymphoma regions. However, this new methodology will require prospective validation in sufficiently large patient cohorts.

Among the included studies, there were mainly two different approaches to define the optimal TMTV cut-off value as a predictor of survival: X-tile analysis, receiver operating curve (ROC) analysis, or both. X-tile analysis is the primary approach for reliable cut-point determination. This method creates separate training and validation data sets, improving the robustness of the analysis [53]. In the studies included in this meta-analysis, ROC was widely used. This method defines the optimal cut-off point as the value whose sensitivity and specificity are closest to the value of the area under the ROC curve, and for which the absolute value of the difference between the sensitivity and specificity values is minimal. This method is recommended for finding the true cut-off point [54]. Meignan et al. [18] used another restricted cubic spline to define the optimal TMTV cut-off point. Splines are used to model the relationship between TMTV as a continuous variable and survival time, but their contribution to optimal cut-off point definition is minimal. Subgroup analyses based on different MTV cut-off values demonstrated that patients with a high TMTV had shorter PFS and OS than those with a low TMTV.

A major strength of this meta-analysis is that it complied with the Preferred Reporting Items for Systematic Reviews and Meta-Analyses (PRISMA) guidelines [55]. In addition, we extracted the maximum information from the included studies by a thorough qualitative review and quantitative meta-analysis.

Our study also has some limitations. Firstly, nearly all of the included studies were retrospective, which may result in confounding and detection bias. Secondly, patients with different types of lymphoma were treated with different therapeutic regimes. Thirdly, PET scans were performed using scanners of different generations, which may potentially affect the calculation of the SUV and therefore, of TMTV and TLG as well. Similarly, the FDG uptake times were difficult to standardize. Based on all of the above, the clinical heterogeneity of the included studies could be an issue. Finally, our meta-analysis was based on data from published trials, and we did not obtain individual patient data.

### Conclusions

Our meta-analysis suggests that high baseline metabolic tumor volumes or total lesion glycolysis measured by FDG-PET/CT predict significantly worse overall survival and progression-free survival in patients with lymphoma. Therefore, TMTV and TLG may serve as new prognostic biomarkers. In view of our findings, future clinical trials with patients with different types of lymphoma are warranted to determine whether these novel findings can be integrated into various prognostic models, with the goal of achieving better risk stratification and treatment selection.

## Supporting information

S1 Text PRISMA checklist. (DOC)

S1 Table Risk of bias assessment. (DOCX)

S2 Table Characteristics of studies included in the meta-analysis. (DOCX)

S3 Table Excluded studies. (DOCX)

S1 Appendix Search strategy. (DOCX)
