# Supplementary material for: Prognostic value of baseline metabolic tumor volume and total lesion glycolysis in patients with lymphoma: A meta-analysis
Source: PLoS One. 2019 Jan 9;14(1):e0210224. doi: 10.1371/journal.pone.0210224 (PMC6326501; doi:10.1371/journal.pone.0210224)
Supplement: S1 Table — (DOCX) [file pone.0210224.s002.docx]

| **S1 Table. The assessment of the risk of bias in each Cohort study using the Newcastle–Ottawa scale** | | | | | | | | | | | | | |
| --- | --- | --- | --- | --- | --- | --- | --- | --- | --- | --- | --- | --- | --- |
|  |  | **Selection (0–4)** | |  |  | **Comparability (0–2)** | |  | **Outcome (0–3)** | | |  |  |
| **Study** | **REC** | **SNEC** | **AE** | **DO** |  | **SC** | **AF** |  | **AO** | **FU** | **AFU** |  | **Total** |
| Song et al.^a^ | 1 | 1 | 1 | 1 |  | 0 | 1 |  | 1 | 1 | 1 |  | 8 |
| Manohar et al. | 1 | 1 | 1 | 1 |  | 0 | 0 |  | 1 | 1 | 0 |  | 6 |
| Kim et al. | 1 | 1 | 1 | 1 |  | 1 | 1 |  | 1 | 0 | 1 |  | 8 |
| Oh et al. | 1 | 1 | 1 | 1 |  | 1 | 0 |  | 1 | 0 | 0 |  | 6 |
| Song et al. | 1 | 1 | 1 | 1 |  | 1 | 0 |  | 1 | 1 | 0 |  | 7 |
| Kim et al. | 1 | 1 | 1 | 1 |  | 0 | 0 |  | 1 | 0 | 0 |  | 5 |
| Esfahani et al. | 1 | 1 | 1 | 1 |  | 0 | 0 |  | 1 | 1 | 1 |  | 7 |
| Sasanelli et al. | 1 | 1 | 1 | 1 |  | 1 | 1 |  | 1 | 1 | 1 |  | 9 |
| Gallicchio et al. | 1 | 1 | 1 | 1 |  | 0 | 0 |  | 0 | 0 | 1 |  | 5 |
| Kim et al. | 0 | 1 | 1 | 1 |  | 0 | 0 |  | 1 | 0 | 1 |  | 5 |
| Adams et al. | 1 | 1 | 1 | 1 |  | 1 | 1 |  | 1 | 1 | 1 |  | 9 |
| Schoder et al. | 1 | 1 | 1 | 1 |  | 1 | 0 |  | 0 | 1 | 0 |  | 6 |
| Kanoun et al. | 1 | 1 | 1 | 1 |  | 1 | 0 |  | 1 | 1 | 1 |  | 8 |
| Mikhaeel et al. | 1 | 1 | 1 | 1 |  | 1 | 0 |  | 1 | 1 | 1 |  | 8 |
| Cottereau et al. | 1 | 1 | 1 | 1 |  | 1 | 1 |  | 1 | 1 | 1 |  | 9 |
| Zhou et al. | 1 | 1 | 1 | 1 |  | 1 | 0 |  | 1 | 1 | 1 |  | 8 |
| Song et al.^b^ | 0 | 1 | 1 | 1 |  | 0 | 0 |  | 1 | 1 | 1 |  | 6 |
| Cottereau et al. | 1 | 1 | 1 | 1 |  | 1 | 1 |  | 1 | 0 | 1 |  | 8 |
| Meignan et al. | 1 | 1 | 1 | 1 |  | 1 | 1 |  | 1 | 1 | 1 |  | 9 |
| Chang et al. | 1 | 1 | 1 | 1 |  | 0 | 0 |  | 1 | 0 | 1 |  | 6 |
| Chang et al. | 1 | 1 | 1 | 1 |  | 1 | 0 |  | 1 | 0 | 1 |  | 7 |
| Kesavan et al. | 1 | 1 | 1 | 1 |  | 1 | 0 |  | 1 | 1 | 0 |  | 7 |
| Song et al. | 0 | 0 | 1 | 1 |  | 1 | 0 |  | 1 | 0 | 1 |  | 5 |
| Cottereau et al. | 1 | 1 | 1 | 1 |  | 1 | 1 |  | 1 | 1 | 1 |  | 9 |
| Pak et al. | 1 | 1 | 1 | 1 |  | 1 | 0 |  | 1 | 0 | 1 |  | 7 |

“1” indicates that the study has satisfied the item and “0” indications the opposite. REC representativeness of the exposed cohort, SNEC selection of the non-exposed cohort, AE ascertainment of exposure, DO demonstration that outcome of interest was not present at start of study, SC study controls for stage, IPI, AF study controls for any additional factors (Chemotherapy, radiotherapy), AO assessment of outcome, FU follow-up long enough (36 M) for outcomes to occur, AFU adequacy of follow-up of cohorts (≥90 %)

**The assessment of the risk of bias in each Cohort study using the Newcastle–Ottawa scale**

**Selection**

1) Representativeness of the exposed cohort

a) truly representative of the source population **(one star)**

b) somewhat representative of the source population **(one star)**

c) selected group of users eg nurses, volunteers

d) no description of the derivation of the cohort

2) Selection of the non exposed cohort

a) drawn from the same community as the exposed cohort **(one star)**

b) drawn from a different source

c) no description of the derivation of the non exposed cohort

3) Ascertainment of exposure

a) secure record (e.g surgical records) **(one star)**

b) directly measured by the study **(one star)**

c) structured interview **(one star)**

d) Written self-report

e) No description/ not clear

4) Demonstration that outcome of interest was not present at start of study

a) yes **(one star)**

b) no

**Comparability**

1) Do main analyses of observational data control for confounders?

a) yes **(one star)**

b) no

c) information not provided/ not clear

**Outcome**

1) Assessment of outcome

a) independent blind assessment **(one star)**

b) record linkage **(one star)**

c) self report

d) no description

2) Was follow-up long enough for outcomes to occur

a) yes (follow up exceeded 3 years) **(one star)**

b) no

3) Adequacy of follow up of cohorts

a) complete follow up - all subjects accounted for **(one star)**

b) subjects lost to follow up unlikely to introduce bias - number lost less than or equal to 25% follow up, or description provided of those lost) **(one star)**

c) follow up rate less than 80% (select an adequate %) and no description of those lost

d) no statement
