# Supplementary material for: Prognostic value of baseline metabolic tumor volume and total lesion glycolysis in patients with lymphoma: A meta-analysis
Source: PLoS One. 2019 Jan 9;14(1):e0210224. doi: 10.1371/journal.pone.0210224 (PMC6326501; doi:10.1371/journal.pone.0210224)
Supplement: S2 Table — (DOCX) [file pone.0210224.s003.docx]

| **Table 2. Characteristics of studies included in the meta-analysis.** | | | | | | | | | | | | |
| --- | --- | --- | --- | --- | --- | --- | --- | --- | --- | --- | --- | --- |
|  |  |  |  |  |  | **Tumor Volume Parameters（MTV/TLG）** | | | | | **Cut-off values** | |
| Study | Year | Country | Study Design | Modality | Type of Lymphoma | Threshold (%) | Median MTV (cm^3^) | Range (cm^3^) | Median TLG | Range | MTV (cm^3^) | TLG |
| Song et al.^a^ | 2012 | Korea | R | PET/CT | DLBCL | ≥2.5 | 198.1 | 4.5–1,990.7 | NR | NR | 220 | NR |
| Manohar et al. | 2012 | India | R | PET/CT | NHL§ | Background-level † | 957 | 56-7332 | 5356 | 434–34783 | 416 | 3340 |
| Kim et al. | 2013 | Korea | R | PET/CT | DLBCL | Various‡ | NR | NR | 415.5 | 4.74-1498.6 | NR | 415.5 |
| Oh et al. | 2013 | Korea | R | PET/CT | DLBCL | ≥2.5 | 156.89 | 4.5–1990.69 | NR | NR | 65.975 | NR |
| Song et al. | 2013 | Korea | R | PET/CT | HL | ≥2.5 | 142.6 | 6.1–587.2 | NR | NR | 198 | NR |
| Kim et al. | 2013 | Korea | R | PET/CT | ENKTL | NR | 10.7 | 9.5-14.9 | 46.9 | 21.0-139.8 | 14.4 | 52.7 |
| Esfahani et al. | 2013 | USA | R | PET/CT | DLBCL | 50 | 379.16 | NR | 704.77 | NR | 379.16 | 704.77 |
| Sasanelli et al. | 2014 | France | R | PET/CT | DLBCL | 41 | 315 | 4-2,654 | 2974 | 14-21908 | 550 | 4,576 |
| Gallicchio et al. | 2014 | Italy | R | PET/CT | DLBCL | 42 | 43 | 2–340 | 596.9 | 110–2552 | 16.1 | 589.5 |
| Kim et al. | 2014 | Korea | R | PET/CT | DLBCL | ≥2.5 | 130.7 | NR | NR | NR | 130.7 | NR |
| Adams et al. | 2015 | Netherlands | R | PET/CT | DLBCL | 40 | 272 | 6-2,454 | 2955.4 | 13.4-23321.5 | 272 | 2955.4 |
| Schoder et al. | 2015 | USA | P | PET/CT | DLBCL | Various‡ | 226 | 9-3,453 | NR | NR | NR | NR |
| Kanoun et al. | 2015 | France | R | PET/CT | HL | Various‡ | 160 | 0-1544 | NR | NR | 313 | NR |
| Mikhaeel et al. | 2016 | UK | R | PET/CT | DLBCL | 41 | 595 | 2-7,360 | 4669.52 | 5.69-36570 | 396 | 4541 |
| Cottereau et al. | 2016 | France | R | PET/CT | DLBCL | 41 | 320 | IQR: 106-668 | 3677 | IQR: 1066-6096 | 300 | 3904 |
| Zhou et al. | 2016 | China | R | PET/CT | DLBCL | Background-level † | 50.7 | IQR: 17-151 | 497.3 | IQR: 104-1452 | PFS: 70 OS: 78 | PFS: 827 OS: 726 |
| Song et al.^b^ | 2016 | Korea | R | PET/CT | DLBCL | ≥2.5 | 526.8 | 15.2–3549.2 | NR | NR | 601.2 | NR |
| Cottereau et al. | 2016 | France | R | PET/CT | PTCL | 41 | 224 | 3-3,824 | 1155 | 19–20 800 | 230 | 1068 |
| Meignan et al. | 2016 | France | R | PET/CT | FL 1-3a | 41 | 297 | IQR: 135-567 | NR | NR | 510 | NR |
| Chang et al. | 2017 | China | R | PET/CT | ENKTL | 40 | 11.2 | 0.8–238.8 | 46.4 | 3.1–1858.1 | 16.1 | 44.7 |
| Chang et al. | 2017 | Taiwan | R | PET/CT | DLBCL | ≥2.5 | 550.4* | NR | 3533.2* | NR | 165.4 | 1204.9 |
| Kesavan et al. | 2017 | Australia | P | PET/CT | FL | 41 | 510 | NR | NR | NR | 510 | NR |
| Song et al. | 2017 | Korea | R | PET/CT | ENKTL | ≥2.5 | 36.2 | 5.1-1164.9 | NR | NR | 94.2 | NR |
| Cottereau et al. | 2018 | France | P | PET/CT | HL | 41 | 67 | IQR: 32-114 | 332 | NR | 147 | 495 |
| Pak et al. | 2018 | Korea | R | PET/CT | ENKTL | 40 | NR | NR | NR | NR | 7 | 45.8 |

PET/CT, positron emission tomography/computed tomography; MTV, metabolic tumor volume; TLG, total lesion glycolysis; NOS, the Newcastle-Ottawa-Scale; DLBCL, diffuse large B cell lymphoma; HL, hodgkin lymphoma; NHL, non-hodgkin’s lymphoma; ENKTL, extranodal natural killer/T cell lymphoma; PTCL, peripheral T-cell lymphoma; FL, follicular Lymphoma; R, retrospective; P, prospective; NR, not reported; IQR, interquartile range; N/A, not applicable;

‡ tested various proposed thresholds, including 41%;

† MTV was measured by setting the tumor marginal threshold of liver SUVmean plus 3SDs. SUVmean in liver was calculated in a standard-sized ROI of 3cm in diameter;

§ Of the 51 patients, 39 (77%) had DLBCL, 8 had anaplastic large T-cell lymphoma and 4 had high-grade peripheral T-cell lymphoma.

a, In staged II and III patients without extranodal site involvement; b, In patients with bone marrow involvement of lymphoma.
