# Supplementary material for: Prognostic value of baseline metabolic tumor volume and total lesion glycolysis in patients with lymphoma: A meta-analysis
Source: PLoS One. 2019 Jan 9;14(1):e0210224. doi: 10.1371/journal.pone.0210224 (PMC6326501; doi:10.1371/journal.pone.0210224)
Supplement: S3 Table — (DOCX) [file pone.0210224.s004.docx]

| Excluded studies | Reasons |
| --- | --- |
| Mol Imaging Radionucl Ther 2017;26:83-92 DOI:10.4274/mirt.48658 | **Primary Gastrointestinal Extranodal  Lymphomas** |
| Cancers 2015, 7, 287-304; doi:10.3390/cancers7010287 | **Pediatric Lymphoma** |
| Radiation Oncology 2012, 7:5 | **No hazard ratio provided** |
| Theranostics 2013, Vol. 3, Issue 10 | Review |
| Mol Imaging Biol (2011) 13:785Y792 | **No hazard ratio provided** |
| ClinCancerRes;22(15);3801–9. | **Multiple publications** |
| Blood. 2015;126(8):950-956 | **primary mediastinal large B-cell lymphoma** |
| Clin Cancer Res; 23(8); 1981–7. | **Other topic-Multiple Myeloma** |
| SeminNuclMed48:50–66 | Review |
| Journal of Clinical Oncology, Vol 34, No 30, 2016: pp 3591-3594 | EDITORIAL |
| J Nucl Med 2013; 54:1518–1527 | Review |
| Eur J Nucl Med Mol Imaging. 2018 Sep;45(10):1672-1679 | **Multiple publications** |
| Cancer Sci 106 (2015) 186–193 | relapsed/refractory DLBCL |
| BMC Cancer (2015) 15:198 | Other topic-interim PET/CT |
| Am. J. Hematol. 90:499–503 | Other topic-interim PET/CT |
| J Nucl Med 2012; 53:1829–1835 | **Other topic-Multiple Myeloma** |
| Eur J Nucl Med Mol Imaging (2010) 37:494–504 | **No hazard ratio provided** |
| Med Oncol (2015) 32:446 | Review- meta-analysis |
