# Supplementary material for: Prognostic value of baseline metabolic tumor volume and total lesion glycolysis in patients with lymphoma: A meta-analysis
Source: PLoS One. 2019 Jan 9;14(1):e0210224. doi: 10.1371/journal.pone.0210224 (PMC6326501; doi:10.1371/journal.pone.0210224)
Supplement: S1 Appendix — (DOCX) [file pone.0210224.s005.docx]

The electronic search strategy for PubMed was as follows:

Search lymphoma

#1 Search ("lymphoma"[MeSH Terms] OR lymphom*[All Fields] OR lymphoproliferative [All Fields] OR hodgkin*[All Fields] OR non-hodgkin* [All Fields]). 278954

Search PET CT

#2 Search ("Tomography, emission-computed"[MeSH Terms] OR ("positron emission tomograpy"[MeSH Terms]) OR (computed [All Fields] AND tomograph*[All Fields])). 563001

#3 Search (prognos* OR predict* OR surviv* OR overall survival* OR recurrence* OR progress*). 4030483

#4 Search (((("lymphoma"[MeSH Terms] OR lymphom*[All Fields] OR lymphoproliferative [All Fields] OR hodgkin*[All Fields] OR non-hodgkin* [All Fields]))) AND (("Tomography, emission-computed"[MeSH Terms] OR ("positron emission tomograpy"[MeSH Terms]) OR (computed [All Fields] AND tomograph*[All Fields])))) AND ((prognos* OR predict* OR surviv* OR overall survival* OR recurrence* OR progress*)). 5045

The electronic search strategy for EMBASE was:

#1 positron emission tomography/computed tomography OR PET/CT OR positron emission tomography-computed tomography OR PET-CT OR fuorodeoxyglucose OR FDG OR 18F-FDG OR 18FDG OR FDG-F18 {including related terms} 10018

#2 (lymphoma).mp. [mp=title, abstract, heading word, drug trade name, original title, device manufacturer, drug manufacturer, device trade name, keyword, floating subheading word] 312259

#3 (positron emission tomography computed tomography.mp. or PET/CT or positron emission tomography.mp. or fluorodeoxyglucose.mp. or FDG.mp.) and (lymphoma).mp. [mp=title, abstract, heading word, drug trade name, original title, device manufacturer, drug manufacturer, device trade name, keyword, floating subheading word] 5042
